# Inference of transcriptome signatures of *Escherichia coli* in long-term stationary phase

**DOI:** 10.1038/s41598-023-32525-4

**Published:** 2023-04-06

**Authors:** Sotaro Takano, Hiromi Takahashi, Yoshie Yama, Ryo Miyazaki, Chikara Furusawa, Saburo Tsuru

**Affiliations:** 1 Bioproduction Research Institute, National Institute of Advanced Industrial Science and Technology (AIST), Tsukuba, Japan; 2 Graduate School of Information Science and Technology, Osaka University, Suita, Osaka Japan; 3 Computational Bio Big Data Open Innovation Laboratory (CBBD-OIL), AIST, Tokyo, Japan; 4 Graduate School of Science, Universal Biology Institute, The University of Tokyo, Tokyo, Japan; 5 Center for Biosystem Dynamics Research, RIKEN, Kobe, Japan; 6 Present Address: International Center for Materials Nanoarchitectonics (NIMS), Research Center for Macromolecules and Biomaterials, Tsukuba, Japan

**Keywords:** Functional clustering, Microarrays, Transcriptomics, Bacterial physiology, Bacterial transcription

## Abstract

“Non-growing” is a dominant life form of microorganisms in nature, where available nutrients and resources are limited. In laboratory culture systems, *Escherichia coli* can survive for years under starvation, denoted as long-term stationary phase, where a small fraction of cells manages to survive by recycling resources released from nonviable cells. Although the physiology by which viable cells in long-term stationary phase adapt to prolonged starvation is of great interest, their genome-wide response has not been fully understood. In this study, we analyzed transcriptional profiles of cells exposed to the supernatant of 30-day long-term stationary phase culture and found that their transcriptome profiles displayed several similar responses to those of cells in the 16-h short-term stationary phase. Nevertheless, our results revealed that cells in long-term stationary phase supernatant exhibit higher expressions of stress-response genes such as phage shock proteins (*psp*), and lower expressions of growth-related genes such as ribosomal proteins than those in the short-term stationary phase. We confirmed that the mutant lacking the *psp* operon showed lower survival and growth rate in the long-term stationary phase culture. This study identified transcriptional responses for stress-resistant physiology in the long-term stationary phase environment.

## Introduction

The physiology of growing microorganisms has been quantitatively characterized based on stable and reproducible culture systems since the 1940s^1^. The quantitative approaches have steadily revealed fundamental principles in exponentially growing microorganisms, such as strong dependence of growth rates on available nutrients or cellular macromolecular compositions (e.g., ribosomes, DNA, and RNA)^2–4^. Expression profiles of genes responsible for these interrelations have also been characterized^5–7^, enabling us to understand the physiology of growing cells at the molecular-level mechanisms. However, most prokaryotes in nature live in nutrient-poor environments, such as deep biospheres, where they exhibit slow- or non-growing states^8^. The physiology of such microbial cells has been appreciated as a general topic^9,10^, yet our understanding of how they survive in such nutrient-poor conditions that provide marginal energy for cellular growth or maintenance of basic cellular functions has remained rudimentary. To systematically understand the cellular physiology of starved microbes, key biological processes for their survival should be characterized based on quantitative measurements.

Microorganisms usually retain viability (i.e., the ability to regrow after the nutrients are supplied) during long-term periods of starvation^8–10^. In a laboratory culture system, *Escherichia coli* maintains viability for years after the exhaustion of nutrients^11^. The cells initially grow exponentially in a fresh nutrient medium, but growth is stopped after the consumption of nutrients, where cells enter short-term stationary phase. A majority of the cells die after the short-term stationary phase, but a small fraction of cells (0.1–0.01%) can continue surviving for months or years, which is termed as long-term stationary phase^11^. Especially in the case of carbon starvation in a synthetic minimal medium, the survival kinetics and rates of starved *E. coli* cells are quite reproducible, which enables an understanding of the long-term survival mechanisms based on quantitative measurements^12–14^.

An important finding from these studies on carbon-starved *E. coli* cells is that cellular death and growth are strongly affected by nutrients released from dead cells^13,14^. A theoretical study also demonstrated that the constant survival in long-term stationary phase is accomplished by recycling dead cells and supported by the regulation of the recycling by population density such that they recover their viability when viable cells decrease^14^. However, molecular functions or biological processes uniquely activated in the viable population in long-term stationary phase are still unknown, which impedes further understanding of the long-term survival mechanism regarding the physiological state of viable cells. The viable population in long-term stationary phase can grow by utilizing chemicals released from starved cells^14^, and thus it is expected that growth-related cellular functions in the viable population can be activated by those molecules. On the other hand, the population growth rate in long-term stationary phase is very low compared to that in exponential phase^14^, and thus the cells in long-term stationary phase should exhibit different physiology from those in exponential phase. Direct quantification of the transcriptome in long-term stationary phase culture would be one possible approach to explore the global transcriptional response occurring in prolonged starvation^15^, but it is quite difficult to analyze the transcriptome profile specifically in a small number of viable cells (0.1–0.01%) apart from a vast majority of the nonviable population. Extracting a sufficient amount of molecules from such a small fraction is another technical challenge for any omics approach.

Given that survival in long-term stationary phase relies on nutrients released from dead cells to the culture^13,14^, the physiology of the survivors can be inferred by investigating the cellular response to the supernatant of that cell culture. Therefore, in this study, we analyzed the transcription of over 3700 genes of *E. coli* exposed to the supernatant of long-term stationary phase to forecast genome-wide responses under a prolonged starvation environment. We compared the transcription profile to those of cells in exponential growth or short-term stationary phases, and found distinct gene expression patterns in each condition. The deletion of one highly expressed family protein in the long-term stationary phase environment exhibited lower survival and growth rate under that condition.

## Results

### Experimental design

**Figure 1:**
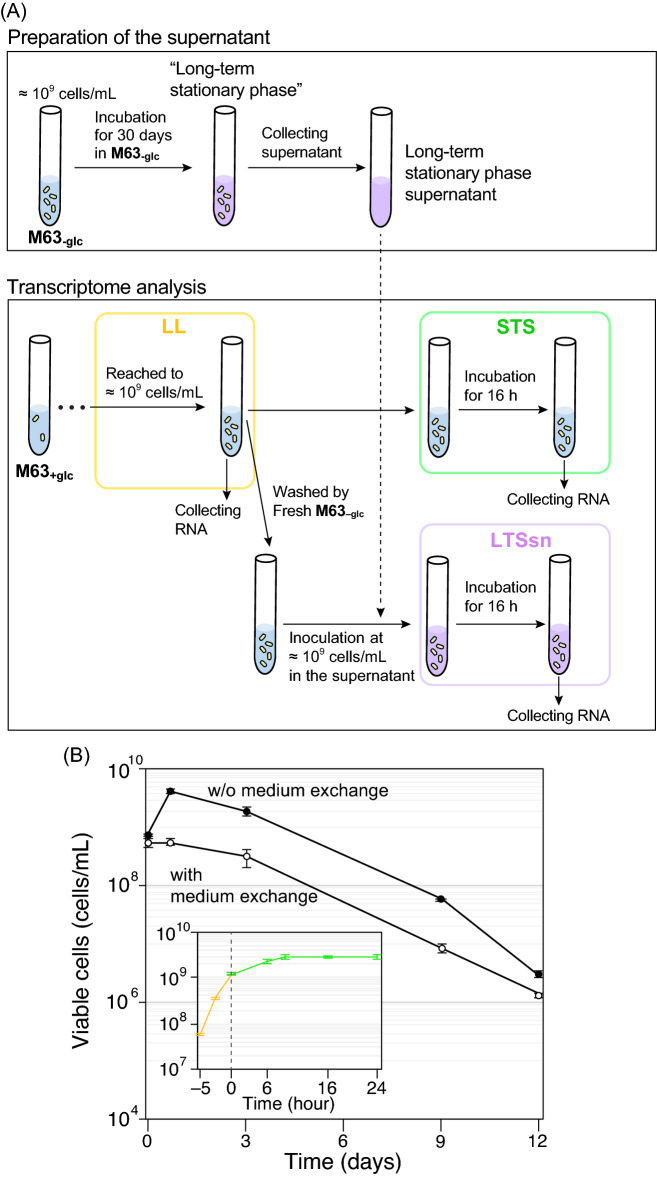
Experimental design for the transcriptome analysis. (**A**) The design for collecting RNA and preparing the supernatant. For the preparation of the supernatant from the long-term stationary phase culture, *E. coli* cells were grown in M63_–glc_ for 30 days, and the supernatant was collected (see “Methods”). For collecting RNA samples, we first cultured *E. coli* cells in M63_+glc_ for 20 h and collected the samples for RNA extraction after the population reached to 10^9^ cells/mL (labeled as LL). Then, this culture was immediately divided into two aliquots. One divided aliquot from the LL phase culture was further incubated in the same condition without medium exchange, which eventually entered short-term stationary phase. We collected the samples, labeled as STS, 16 h after inoculation. To prepare the LTSsn samples, we washed the other aliquot with fresh M63_–glc_ and inoculated it into the long-term stationary phase supernatant. We collected the samples 16 h after medium exchange. (**B**) Temporal changes in the number of viable cells with or without medium exchange (white and black circles, respectively). An inset shows the growth curve of the cells from the LL phase (yellow line) to the short-term stationary phase (green line). The sample preparation was performed according to the procedure explained in panel (**A**). The time point of division into two aliquots was set at 0 h in the figure. The number of viable cells was estimated by the CFUs. Error bars indicate standard deviations of biological triplicates.

The experimental design for investigating gene expression profiles in three different conditions is shown in Fig. 1A. In each condition, *E. coli* was first grown in a glucose-rich synthetic medium (M63 medium with 22.2 mM glucose, designated as M63_+glc_) for 20 h until late-log phase (~ 10^9^ cells/mL). We designated this cell culture as “LL”. We divided the LL culture into two aliquots and further incubated one for 16 h without any medium exchange (then, this culture reached the plateau) and designated it as “STS (cell cultures reached the short-term stationary phase)” (Fig. 1B, inset). The other aliquot was washed with M63 medium without glucose (designated as M63_–glc_, hereafter) three times to eliminate the remaining glucose and resuspended with a supernatant of long-term stationary phase (designated as LTS) and incubated for 16 h. We called the culture with medium exchange as LTSsn. As the incubation time increased beyond 16 h, the number of viable cells (i.e. the cells that can regrow after being transferred to fresh M63_+glc_, see “Methods”) gradually decreased regardless of medium exchange (Fig. 1B). We focused on the transcriptional response specific to a viable population; thus, contamination of dead cells in the cultures was not ideal for the transcriptome analysis. The results of the viability assays by CFU (Colony Forming Unit) revealed that 16 h of incubation after entering into each condition (i.e., the timing at which divided into two aliquots) did not reduce the number of viable cells regardless of medium exchange (Fig. 1B); thus, we collected samples from both of the cell cultures for RNA isolation after 16 h from transferring to each condition. In addition, a 16-h incubation period in M63_+glc_ was sufficient to see the halt of population growth (Fig. 1B, inset). Thus, collecting the samples at this time point should be sufficient for analyzing the physiological differences between the LL and STS samples.

### Expression profiles in LTSsn and STS are similarly grouped together

**Figure 2:**
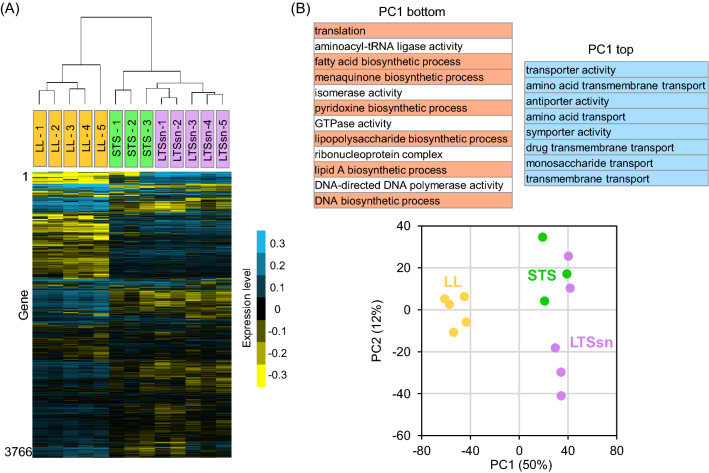
Comprehensive analysis of gene expression data. (**A**) Heatmap of the expression levels in 3,766 genes in the late-log phase culture (LL, n = 5), the short-term stationary phase culture (STS, n = 3), or the long-term stationary phase supernatant (LTSsn, n = 5). In each gene, the expression data were normalized to the mean value of all samples. We performed clustering both by genes and arrays using the average linkage method based on the Euclidean distance among the samples or genes. (**B**) Plots of PCA scores from the analysis using all analyzed samples. Plots indicate scores of individual samples in each condition. The percentage displayed on each axis is the rate of the contribution of PC1 or PC2. GO terms that have significant positive or negative contributions are displayed as PC1 top or bottom groups. GO terms associated with, “transport” and “biosynthetic process” are colored red and blue. All extracted gene sets are shown in Supplementary Data S3.

Then, we extracted RNA from the collected samples and performed a DNA microarray analysis to explore gene expression profiles. We first performed hierarchical clustering for all analyzed samples across all genes to compare the similarity in global transcriptional profiles among three conditions. We found that LL showed different gene expression profiles from the other two conditions (Fig. 2A). Principal component analysis (PCA) also showed a similar tendency in the transcriptional profile: LTSsn and STS are clustered, whereas LL is distinct (Fig. 2B). In particular, the first principal component, PC1, which accounted for 50% of the total variance in the dataset, was found to explain the difference between the two clusters well.

### Screening of the differentially expressed gene sets in LTSsn and STS compared to LL

We characterized functional gene sets that were differentially expressed in LTSsn and STS compared to LL. We performed a hypergeometric test and statistically screened functional gene sets that highly contribute to PC1. We used GO terms for categorizing genes by their functions^16^ (Supplementary Data S1) and focused on the difference in the cellular processes; gene sets classified as child classes or parts of “biological processes (GO:0008150)” and “molecular functions (GO:0003674)” (Fig. 2B). There were two types of screened gene sets: PC1 top and PC1 bottom (Supplementary Fig. S1). Gene sets screened as “PC1 top” included genes positively correlated to PC1 scores in their expression levels (Supplementary Fig. S1). For instance, *gltK,* a member of “transporter activity (GO:0005215)” in the “PC1 top” group, showed a strong positive correlation with the PC1 scores (Supplementary Fig. S1), and the level of expression of this gene was higher in LTSsn and STS (Supplementary Fig. S1). In contrast, the expression levels in the “PC1 bottom” group were negatively correlated with the PC1 scores. *rplV*, which is included in “translation (GO:0006412)” in the “PC1 bottom” group, showed strong negative loading for PC1 (Supplementary Fig. S1), and LL samples showed higher expression levels than the other two samples (Supplementary Fig. S1).

The “PC1 top” gene sets were mostly associated with transport activity (i.e., child classes or) parts of the “transporter activity (GO:0005215)” or “transport (GO:0006810)”), whereas the “PC1 bottom” were occupied by biosynthetic gene sets (i.e., child classes or parts of the “biosynthetic process (GO:0009058)”) (Fig. 2B and Supplementary Data S2). Therefore, higher expression levels of transport genes and lower expression levels of biosynthesis genes would be major transcriptional changes in LTSsn and STS compared to LL. This tendency was confirmed by comparing the average transcripts levels of all gene members in “biosynthetic process (GO:0009058)” and “transport (GO:0006810)” (Supplementary Fig. S2). To check whether the transcriptional changes from LL to STS exhibited a similar tendency to those observed in a previous study, we performed Gene Set Enrichment Analysis (GSEA)^17^ for transcriptome in MG1655 cells (i.e., a parental strain of MDS42) in exponential or typical stationary phase (designated as short-term stationary phase, hereafter)^18^. We screened the functional gene sets (GO terms) that were differentially expressed between exponential and short-term stationary phases in the previous study, and compared them with those screened by the comparison between LL and STS in our study. We found similar transcriptional changes between our study and the previous study (Fig. S3) in terms of lower expression in biosynthetic process (e.g., “translation (GO:0006412)”) and energy metabolism (e.g., “ATP biosynthetic process (GO:0006754)”) and higher expression in amino-acid metabolism (e.g., “arginine metabolic process (GO:0006525)”), suggesting that transcriptional changes in those gene sets are commonly observed at the transition from exponential to short-term stationary phase. On the other hand, we also found a difference in screened gene sets associated with transport function. For instance, “dipeptide transport (GO:0042938)” was screened as a higher expressed category in the short-term stationary phase only in the case of Baptista et al.^18^ (Fig. S3). Whereas, “carbohydrate transport (GO:0008643)”, which is more highly expressed in STS than LL in our study (Fig. S3), averagely exhibited lower expression in stationary phase than exponential phase in the previous study (Fig. S3). When we focus on the transcriptional changes in individual genes in this functional category, we found that the regulation of the carbohydrate transport genes varies depending on target substrates (Fig. S4). We reasoned that the difference in transcriptional changes in those transport genes was mainly due to the difference in the medium and culture condition of the bacterial cells between the two studies (see “Discussion”).

### Comparison of expression profiles between LTSsn and STS

**Figure 3:**
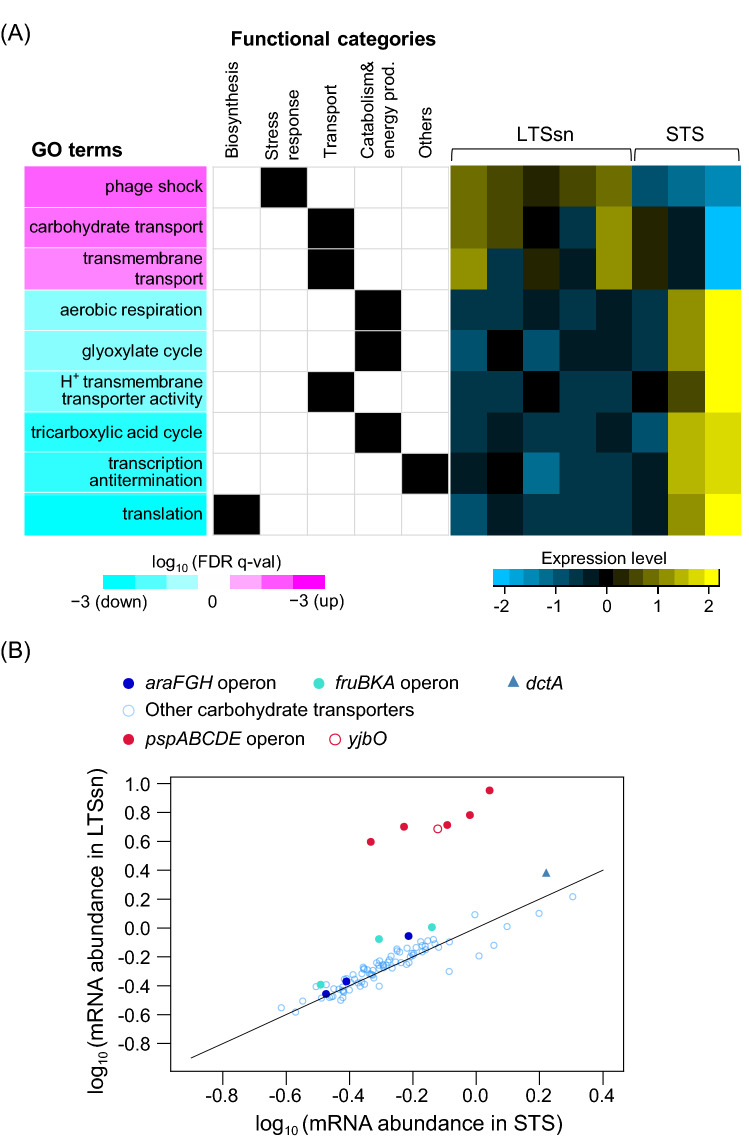
Differentially expressed gene categories in LTSsn compared to STS. (**A**) Representative differentially expressed gene categories extracted by GSEA (False Discovery Rate (FDR), q-value < 0.05). Statistical significance levels (FDR, q-value) are shown as heatmaps. Magenta and cyan in the heat map mean that the expression level of each gene set is higher or lower in LTSsn than in STS, respectively. These screened gene sets were further grouped into five large categories: (1) biosynthesis (child classes or parts of “biosynthetic process (GO:0009058)”), (2) stress response (child classes or parts of “response to stress (GO:0006950)” and “anti-oxidant activity (GO:0016209)”), (3) transport (child classes and parts of “transport (GO:0006810)” or “transporter activity (GO:0005215)”), (4) catabolism and energy production (child classes and parts of “generation of precursor metabolites and energy” (GO:0006091) or “cellular catabolic process” (GO:0044248)), and (5) other categories. If each GO term was grouped into an indicated functional category, the box was filled with black. A yellow-blue scaled heatmap shows the expression levels in each GO term (i.e., the average gene expression levels of all members in each GO term) in biological replicates (LTSsn: n = 5; STS: n = 3). The values of mRNA signals in each extracted GO category were normalized so that the average and the standard deviation in all replicates are 0 and 1. (**B**) Scatter plots of log10 scaled gene expression levels in “phage shock (GO:0009271)” (red circles) and “carbohydrate transport (GO:0008643)” (blue or cyan circles or triangles) in STS or LTSsn.

To understand cellular responses specific to long-term, but not short-term, stationary phase environment, we screened the functional gene sets (GO terms) differentially expressed between LTSsn and STS by GSEA^17^ and identified 23 gene sets (Supplementary Data S4). Significantly highly expressed genes in LTSsn were classified as “phage shock (GO:0009271)” (Fig. 3A), which are known to maintain membrane integrity under stress conditions to avoid leakage of cytoplasmic contents and the loss of proton motive force^19–21^. Another highly expressed functional category was “transport” such as “transmembrane transport (GO:0055085)” in LTSsn. Especially, “carbohydrate transport (GO:0008643)” showed high enrichment scores in the group of “transport” (Fig. 3A), involving genes responsible for uptake of carbohydrates such as *araFGH* (L-arabinose transporter subunits), *fruBKA* operon (fructose phosphotransferase system), and *dctA* (C_4_-dicarboxylic acids transporter) (Fig. 3B).

To understand the regulatory mechanism of those transcriptional changes, we focused on gene sets grouped by their gene regulatory network (Regulon DB database)^22^ and screened transcriptional regulators that controlled differentially expressed genes between LTSsn and STS by GSEA. 8 transcriptional factors were screened as significantly enriched differentially expressed genes (Fig. S5). Genes controlled by RpoN, PspF, and NtrC showed higher expression in LTSsn. PspF is a regulator of the phage shock protein family^23^, which is screened as a highly expressed functional category in LTSsn. NtrC also controls gene expression involved in the uptake of carbon metabolites such as putrescine (i.e., *potFGHI*)^24,25^. Those gene sets were also under the control of RpoN^23,26^. On the other hand, gene sets regulated by ArcA, Fur, NrdR, OmpR, and GadE exhibited lower expression in LTSsn than STS, and most of those regulators control gene expressions that are mainly involved in respiration and energy metabolism. ArcA is known as a regulator of genes involved in aerobic respiration^27,28^ such as “tricarboxylic acid (TCA) cycle (GO:0006099)” and “hydrogen ion transmembrane transporter activity (GO:0015078)”. NrdR and Fur are also regulators of *nrdHIEF*^29,30^, which plays a role in electron transfer to the electron transport chain. GadE also controls the expression of proteins involved in aerobic respiration such as cytochrome *o* ubiquinol oxidase (*cyoABCDE* operon)^31^. Overall, those signatures in gene regulation are consistent with the screened functional categories.

### Physiological significance of highly expressed genes in LTSsn

**Figure 4:**
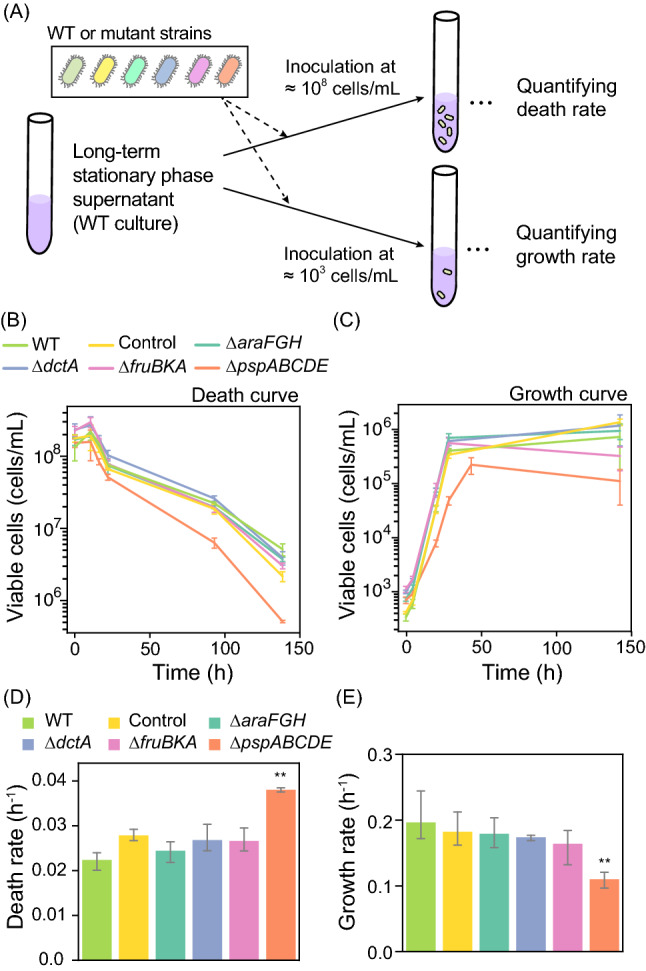
The effect of the deletions of highly expressed genes in LTSsn on survival and growth in the long-term stationary phase supernatant. (**A**) Experimental design for measuring death and growth rate of mutant strains in the long-term stationary phase supernatant. We prepared the supernatant from the WT cell cultures, and inoculated cells of mutant or WT strains. (**B**, **C**) Temporal changes in the number of viable cells in the MDS42 or its mutant strains inoculated into the LSP supernatant at 10^8^ or 10^3^ cells/mL. The number of viable cells was estimated by the CFUs. Error bars indicate standard deviations of biological triplicates. (**D**, **E**) Death and growth rate of knock-out mutants in the long-term stationary phase supernatant. From the population growth or decay curves in panels A and B, we estimated the growth and death rates in each strain. Error bars indicate the standard deviation of biological replicates (n = 3). We performed Dunnett’s test between each of the knock-out mutants (*∆araFGH, ∆dctA, ∆fruBKA,* or *∆pspABCDE*) and a “Control” strain (see “Methods”) (**, p < 0.05).

We next explored whether the genes that were highly expressed in LTSsn such as “phage shock (GO:0009271)” and “carbohydrate transport (GO:0008643)” play a role in *E. coli* survival in LTSsn. We constructed mutants lacking *pspABCDE* operon, *araFGH* operon, *fruBKA* operon, or *dctA*, which are especially highly expressed genes in LTSsn (Fig. 3B), and compared the survival kinetics of those mutants to a control strain (see “Methods”). We prepared the supernatant of long-term stationary phase culture by culturing WT strain for 30 days in M63_–glc_, and cells of the mutants were inoculated at 10^8^ cells/mL (Fig. 4A). All cell cultures exhibited a decrease in the number of viable cells 16 h after inoculation (Fig. 4B). Notably, the *pspABCDE* mutant showed significantly rapid decay in the viability (Fig. 4B, D). We also compared the growth kinetics of those strains in the supernatant of long-term stationary phase culture by inoculating them at 10^3^ cells/mL (Fig. 4A). The *pspABCDE* mutant showed significantly slower growth than its wild-type or control strains while other mutant strains did not (Fig. 4C, E). These results indicate that the expression of phage shock proteins supports survival and growth in the supernatant of long-term stationary phase culture. However, the loss of genes associated with carbohydrate transport did not alter growth and survival kinetics compared to the WT, suggesting that the expression of those gene sets would not be a physiologically significant response but the byproduct of transcriptional regulation in the long-term stationary phase (see “Discussion”).

### Survival and maintenance weighed expression profiles in LTSsn

**Figure 5:**
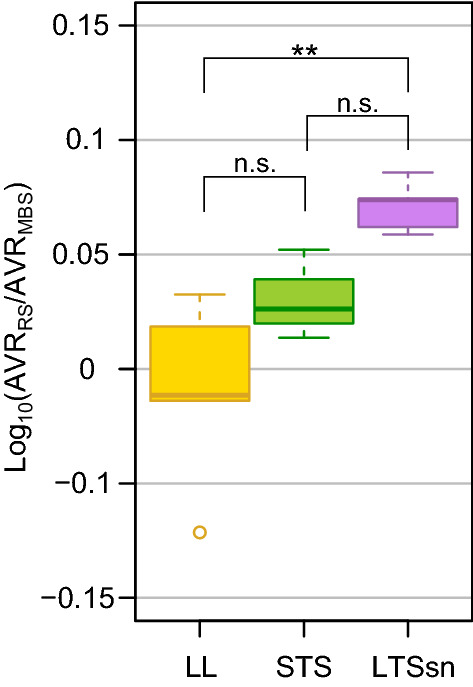
Stress-response weighed expression in LTSsn. log10 scaled ratio between the average mRNA signals of all members in “macromolecule biosynthetic process (GO:0009059),” designated as “AVR_MBS_”, or “response to stress (GO:0006950),” designated as “AVR_RS_”. Error bars indicate the standard deviation of average signals among the biological replicates. Asterisks indicate statistical significance levels according to Tukey’s HSD test (**, p < 0.01; *, p < 0.05).

In contrast to higher expression levels and physiological significance in phage shock protein family, genes in “translation (GO:0006412),”, “aerobic respiration (GO:0009060)” and “tricarboxylic acid (TCA) cycle (GO:0006099),” were lower in LTSsn than in STS (Fig. 3A). These gene sets are associated with protein production and ATP concentration, which are strongly related to cellular growth^6,32^. These results suggest that cells in STS still retain somewhat resource allocation to cell growth, whereas LTSsn exhibits more static and stress/survival-weighed physiology. To explore the balance between growth- and stress/survival-related cellular functions, we compared the ratio of average expression levels between growth-related and stress-response gene sets among LTSsn, STS, and LL. To characterize the balance between growth and stress-response (stress-hedging) at the molecular level, previous studies often used collective expression levels of transcription-, translation-related genes (such as ribosomal proteins) for growth and genes related to various stressful conditions (such as osmotic stress or oxidative stress) for stress-response^32–34^. Following these functional categories in terms of gene ontology, we used GO categories of “macromolecule biosynthetic process (MBS)” (GO:0009059) as a gene set for cell growth, and “response to stress (RS)” (GO:0006950) as a gene set for stress survival (Supplementary Data S3), and then calculated a ratio of their average expression levels (AVR_RS_/AVR_MBS_). The ratio was significantly higher in LTSsn than in LL (p-value < 0.01, Fig. 5), suggesting that the cells in long-term stationary phase environment weighted stress/survival tasks. In contrast, the ratio was lower in STS than in LTSsn, and there was no significant difference between STS and LL, supporting that LTSsn would be in a more stress/survival-weighed physiology than STS.

## Discussion

Our transcriptome analysis revealed a common transcriptional response in short-term stationary phase and stationary phase mimicking long-term stationary phase. Compared to cells in the exponentially growing phase (LL), one of the common major transcriptional changes in STS and LTSsn was a decrease of gene expression for the biosynthesis of fatty acids, lipopolysaccharides, proteins, and vitamins (i.e., menaquinone and pyridoxine) (Fig. 2B and Supplementary Fig. S2).

Especially, the gene sets involved in protein biosynthesis (i.e., translation) screened as a lower expressed functional category in short-term stationary phase compared to exponential phase in the other *E. coli* strain^18^, and down-regulation of this gene set is not specific to our experimental setup. As these biosynthetic gene sets are involved in the synthesis of major cellular components and essential for cellular growth in *E. coli*^35,36^, their low expression levels would be one of the possible mechanisms to explain the non-growing state of cells in both short- and long-term stationary phase, which is a common tendency of bacterial transcriptional response after halting growth^37–41^. We also revealed that the LTSsn and STS showed higher expression levels in genes associated with transport than LL (Fig. 2B and Supplementary Fig. S2). The cultures of LTSsn and STS must contain various chemical compounds released from cells (e.g., carbohydrates, nucleotides, and amino-acids)^42–44^, and a high expression of various transporter genes to be beneficial for cells to import and recycle ambient nutrients for their survival. Furthermore, the importance of the ability to utilize compounds released from dead cells in long-term stationary phase has been previously reported^13,14,42^. Additionally, the scavenging of remaining nutrients is one of the major responses in the short-term stationary phase as previous studies suggested^45,46^. Activation of transport functions would be one of the important mechanisms for constant survival in short- and long-term stationary phases. However, we also found that some of the highly expressed carbohydrate transport genes in STS showed lower expression levels in short-term stationary phase than exponential phase in the previous study (Fig. S4)^18^. When we compared the expression profiles between the previous and our studies, there were mainly three types of transcriptional changes in this category: down-regulated in short-term stationary phase commonly in both studies; up-regulated in short-term stationary phase commonly in both studies; up-regulated only in our study. The genes encoding the glucose phosphotransferase system (e.g., *ptsG* and *ptsH*) exhibited lower expression levels both in STS (our study) or short-term stationary phase (the previous study)^18^, which would correspond to the depletion of glucose (a major carbon source) from the exponentially growing phase. Transport genes responsible for the uptake of rhamnose, melibiose, and mannitol (i.e., *rhaT*, *melB*, and *mtlA*) showed higher expressions in short-term stationary phase in both studies, and utilization of those carbohydrates would be up-regulated in both experimental conditions. On the other hand, genes involved in the transport of allose, maltose, and gluconate (i.e., *alsBAC, malEFG, gntT*, and *gntU*) exhibited higher expressions in short-term stationary phase only in our experiments, and this deviation would reflect the difference in available nutrients during short-term stationary phase between the two studies. Therefore, what type of transport genes were highly expressed in short-term stationary phase would depend on the experimental setup (e.g., medium condition and type of strain).

Although LTSsn has similar transcriptional characteristics to STS, several functional gene sets were differentially expressed between them, and we identified possible transcriptional regulators responsible for those transcriptional changes (Fig. S5). One of the major differences is higher expression levels of protein-synthesis genes (e.g., translation) in STS than in LTSsn. Protein synthesis is an energy-consuming process coupled to cellular growth in bacteria^2,4,47^. Therefore, higher expression levels of those gene sets indicate more growth-weighed physiology in short-term stationary phase than long-term stationary phase. A previous study also demonstrated that *E. coli* cells entering into short-term stationary phase do not completely stop the production of proteins but still produce proteins, albeit at a relatively lower level, at least for about 40 h after growth arrest^48^, supporting that the transcriptional signature of active protein synthesis in STS reflects the functional activity of protein synthesis. Conversely, phage shock protein family, one of the important cell envelope stress response genes^19–21^, was strongly induced in LTSsn (Fig. 3A), suggesting that the physiology of LTSsn would be a more stress-resistant state rather than a reproductive state. We also revealed that the presence of phage shock protein family contributes to survival in long-term stationary phase (Fig. 4). A previous study reported that phage shock protein defective mutants exhibited less survivability in death phase after short-term stationary phase only at alkaline pH conditions and suggest that those gene sets play a crucial role only when cells were exposed to both nutritional and pH stresses^49^. Our results showed that those gene sets are also important for survival at neutral pH conditions in the long-term stationary phase supernatant (we confirmed that pH in the long-term stationary phase supernatant used in this study maintained ≈ 7.0), suggesting that *psp* operons contribute to starvation survival apart from pH stress. It was also reported that the viability of *psp* mutants in starvation declined more rapidly when co-cultured with wild-type cells probably because *psp* defective mutants reduce the ability to utilize limited resources remaining in the medium^49^. In this study, we directly detected a decrease in the growth rate in the long-term stationary phase supernatant by the deletion of *psp* operons (Fig. 4), which also supports the importance of those genes for the utilization of limited nutrients for growth. Given these results, *psp* operons would facilitate survival in long-term stationary phase environment by contributing to not only membrane stress resistance but also the uptake and utilization of limited nutrients.

LTSsn also exhibited higher gene expressions involved in substrate uptake such as carbohydrate transport genes than STS (Fig. 3A). However, our viability and growth assay of mutants showed that those genes would not play a significant role in growth and survival in the long-term stationary phase supernatant (Fig. 4). One possible reason is the small increase in those transcripts in LTSsn compared to STS. Indeed, those transport genes were screened by an enrichment analysis, not by fold-change analysis for individual genes, and most of them exhibited no more than a 1.4-fold increase in LTSsn, which was a quite small change compared to the case of other genes (e.g., *psp* operon) (Fig. 3B). In addition to this, we speculate that the expression of those transport genes would be a byproduct of transcriptional regulation in the starvation condition. It is well known that expressions of carbohydrate transport genes are induced in the presence of their targeting molecules but rapidly repressed by an accumulation of cAMP after catabolizing those molecules (known as catabolite repression). For instance, the expression level of *araFGH* increases within 10 min after the supplementation of arabinose but decreases within 80 min^50^. However, those genes were still highly expressed after 16 h of incubation in LTSsn. If the increase of *araFGH* expression occurs in response to the long-term stationary phase environment and contributes to the uptake and metabolization of arabinose, the expression level of those genes should return to the original level (expression level in LL). One possible scenario for explaining this disorder in transcriptional regulation is that uptake and metabolization of the sugars or subsequent ATP production process stagnate in the long-term stationary phase environment. In that case, accumulation of cAMP and subsequent transcriptional repression of those transport genes does not occur. Given those transcriptions responsible for ATP production (e.g., “aerobic respiration (GO:0009060)” and “tricarboxylic acid (TCA) cycle (GO:0006099),”) are suppressed in LTSsn than STS, it is plausible that imported molecules are not fully converted to ATP, and sufficient amount of cAMP does not accumulate in the cells. Also, induction of phage shock proteins is known to lead to the repression of aerobic respiration and increase of anaerobic respiration and fermentation^51^ through ArcA/ArcB system, suggesting downregulation of energy production processes. Taking all of these transcriptome signatures in LTSsn together, the expression of carbohydrate transporters genes does not fully contribute to the survival or growth probably due to the low metabolic activities of the cells in a long-term stationary phase environment.

In conclusion, our study using DNA microarray characterized the genome-wide response of cells in the environment of long-term stationary phase and screened potentially important cellular processes for long-term survival. Although a detailed gene regulatory network for the long-term stationary phase environment is still unclear, our results propose candidates for such important gene members and pave the way to understand molecular mechanisms for surviving prolonged starvation.

## Methods

### Strains

We used the *E. coli* strain MDS42, which was purchased from Scarab Genomics (Madison, WI, USA), for transcriptome analysis. The MDS42 mutant strains listed in Table 1 were constructed as previously described^52^. In brief, the *tetA-sacB* DNA cassette was amplified from an SJ_XTL219 (purchased from addgene (Watertown, MA, USA)) by PCR with chimeric primers containing homology arms for targeting DNA regions (primer sequences used for PCR are listed in Table 2). These PCR products are used for λ RED-mediated homologous recombination^53^. As a control, we constructed a strain where the *tetA-sacB* cassette was inserted between the *argW* and *ypdJ* genes. The tetracycline resistance (Tc^R^) recombinants were selected by the 1.5% LB agar selection media supplemented with Tetracycline (12.5 μg/mL).

**Table 1:** Bacterial strains used in this study.

Strain Name	Genotype
MDS42	MDS42
Co (control)	MDS42 *argW*<*tetA-sacB*>*ypdJ*
*ΔaraFGH*	MDS42 *ΔaraFGH*::*tetA-sacB*
*ΔdctA*	MDS42 *ΔdctA*::*tetA-sacB*
*ΔfruBKA*	MDS42 *ΔfruBKA*::*tetA-sacB*
*ΔpspABCDE*	MDS42 *ΔpspABCDE*::*tetA-sacB*

The <*tetA-sacB*> designation indicates the insertion of the cassette in an intergenic region of two genes.

**Table 2:** Oligonucleotides and templates used for the construction of mutant strains.

Primer name	Sequence (5′–3′)	Template	Constructed strains
argW_tet	atgacgggtaaaaagtggataaaataattttacccaccggatttttaccctcctaatttttgttgacactctatc	SJ_XTL219	Co (control)
ypdJ_sacB	tattttaagaaactgacaggcctcatcgagtgtgaggctgtatggctctaaaagggaaaactgtccatatgc	SJ_XTL219	Co (control)
yecItsac_Fw	tgcgcaaacacccgcactcggggaagggagtgcgggcataagtgatgagaaaagggaaaactgtccatatgc	SJ_XTL219	*ΔaraFGH*
otsBtsac_Rv	taacagttcagcaggacaatcctgaacgcagaaatcaagaggacaacatttcctaatttttgttgacactctatc	SJ_XTL219	*ΔaraFGH*
yhjKtsac_Fw	tgtcattcgtttttgccctacacaaaacgacactaaagctggagagaacctcctaatttttgttgacactctatc	SJ_XTL219	*ΔdctA*
yhjJtsac_Rv	ggcggttggctatggtgggaaaaaacgctaaattgttgcagaaaaaagcaaaagggaaaactgtccatatgc	SJ_XTL219	*ΔdctA*
fruKtsac_Fw	tcatcaaatgttacaggacaggaaatttctgccctgtaacacaccttttaaaagggaaaactgtccatatgc	SJ_XTL219	*ΔfruBKA*
yeiCtsac_Rv	caggtaccccataaccttacaagacctgtggttttactaaaggacacccttcctaatttttgttgacactctatc	SJ_XTL219	*ΔfruBKA*
pspFtsac_Fw	gtgtgacagaaaaaaaaacggcgcataagcgccgctcatggtgaattcttaaagggaaaactgtccatatgc	SJ_XTL219	*ΔpspABCDE*
ycjMtsac_Rv	gatggcgcgcgtcgacttacaaccttttaactgacagcaggagaggcatatcctaatttttgttgacactctatc	SJ_XTL219	*ΔpspABCDE*

### Culture conditions and RNA sample preparation for DNA microarray

The *E. coli* cells were inoculated into fresh M63 medium (62 mM K_2_HPO_4_, 39 mM KH_2_PO_4_, 15 mM (NH_4_)_2_SO_4_, 2 µM FeSO_4_∙7H_2_O, 200 µM MgSO_4_∙7H_2_O, pH 7.0) with 22 mM glucose from glycerol stock and grown for 20 h at a density of approximately 1 × 10^9^ cells/mL. Cells were then shaken at 160 rpm in a BR-21FP air incubator (Taitec, Saitama, Japan) at 37 °C aerobically. We divided this culture into two aliquots. One aliquot of the grown cultures was further incubated at 37 °C for 16 h to analyze transcriptome in STS. For the transcriptome analysis of the LTSsn, the other aliquot was centrifuged at 7500 × g for 3 min and resuspended by M63 minimal medium without glucose (M63_–glc_). These washing steps were performed three times to eliminate glucose in the culture, and then samples were inoculated into the supernatant of long-term stationary phase (see also Fig. 1A and below). Of note, we did not perform the washing steps in STS samples to exclude the effect on the exposure to M63_–glc_, which would result in different physiology from the typical stationary phase. One might assume that this difference in experimental procedure would lead to the difference in transcriptome between LTS and STS. However, the effect of washing steps is deemed to be very small because more than 100 turnovers of mRNA are expected in 16 h given that the lifetime of mRNA is less than 10 min^54^. To obtain a sufficient amount of mRNA molecules from the culture, we cultured cells at a concentration of > 10^9^ cells/mL in all experimental conditions and collected > 10^9^ cells from each sample. To harvest the samples for GeneChip analysis, the cell cultures were mixed into chilled phenol-ethanol solution (1 g of phenol in 10 mL of ethanol) and centrifuged at 7500 × g for 3 min at 4 °C. After eliminating the supernatants, the cell pellets were frozen by liquid nitrogen and stored at − 80 °C. Total RNA was extracted using a PureLink Mini kit (Thermo Fisher Scientific, Waltham, MA, USA) according to the manufacturer's protocol.

### Microarrays and data normalization

The cDNA synthesis from the purified RNA, fragmentation, labeling, and hybridization of cDNA were performed in accordance with the manufacturer's protocol of Affymetrix (Santa Clara, CA, USA). A high-density DNA microarray that covers the whole genome of *E. coli* W3110 strain was used for analysis. Microarray data were extracted based on the finite hybridization model as previously described^55,56^, and the data sets of genes in which average values were less than − 1.5 pM were eliminated to prevent the effects of noise derived from the small values as described previously^57^. These data sets were further normalized so that all data sets have common statistics on a logarithmic scale^58^. Finally, we used a total of 3,766 genes for the following analysis.

### Computational data analysis

Clustering of genes and arrays according to 3766 transcriptional profiles was performed by Cluster 3.0^59^ using the average linkage method of the Euclidean distance.

A GO term annotation was used for the functional classification of genes in all transcriptome analyses, and the annotation list of *E. coli* strain K-12 was collected from Ecocyc^16^ (http://www.biocyc.org), with slight modifications (Supplementary Data S1). To make 3766-dimensional data to low dimension and visualize simply, we performed PCA and clustering employing R-package^60^ using the correlation matrices of the normalized data sets. If factor loading in the top or bottom 3% among all analyzed genes and more than 0.8 or less than − 0.8, we identified those genes as highly contributing genes to each PC score. For screening GO terms including a significantly large number of genes with high loading-factor, we used a hypergeometric test (p < 0.01) as previously described^61^. For studying differentially expressed gene sets, we conducted Gene Set Enrichment Analysis (GSEA)^17^. Briefly, all 3766 genes were ranked by the difference in the average mRNA level between LTSsn and STS, then an enrichment score of each GO terms (i.e., the degree to which the members of the GO term are enriched at the top or bottom of the ranked gene list) was calculated. We excluded GO terms consisting of less than 6 genes or more than 200 genes for GSEA, PCA, and post hoc analysis.

### Estimation of the growth and death rate

In Fig., the growth and death rate *µ* in each strain was calculated by the following equation.

$${\mu }_{i}=\left|\frac{\mathrm{log}({N}_{i+1}/{N}_{i})}{{t}_{i+1}-{t}_{i}}\right|$$

Here, *N*_*i*_ denotes the number of viable cells at timepoint *i*, *t*_*i*_ denotes the time from the start of experiments to timepoint *i*, and *µ*_*i*_ denotes the growth or death rate from *i* to *i* + 1. For the estimation of the death and growth rate, we used the data of *i* = 3– 5 timepoints (*t*_*i*_ = 22–138.5 h) and *i* = 0–3 timepoints (*t*_*i*_ = 0–28.5 h), respectively. We calculated the average of *µ*_*i*_ over given timepoints and designated it as *µ* for each strain.

### Viability assay

The MDS42 and its mutant strains were first cultured in fresh M63_+glc_ with shaken at 160 rpm at 37 °C aerobically. For the experiments in the long-term stationary phase condition, the cell cultures were washed by M63_–glc_ thrice after the cell concentration of the cultures reached approximately 10^9^ cells/mL. Then, samples were inoculated into the supernatant of long-term stationary phase culture of the MDS42 WT strain. The number of viable cells (cells retaining growth ability) was estimated by colony-forming unit (CFU) in all experiments. Cell cultures incubated in each condition were harvested, diluted, and inoculated onto M63 agar (1.5%) plates supplemented with 0.4% glucose. The duplicate inoculated plates were incubated at 37 °C for approximately 2 days, and the number of colonies was manually counted.

### Preparation of supernatants from long-term stationary phase cultures

We grew *E. coli* MDS42 WT cells in M63_+glc_ for ≈ 20 h at 37 ºC aerobically with shaking at 160 rpm and washed the cells thrice with M63_–glc_, as described above. The glucose-starved cultures were incubated for 30 days at 37 °C aerobically with shaking at 160 rpm. After 30 days of incubation, the cultures were harvested by centrifugation at 7500 × g for 3 min at room temperature. The supernatants were filtered with a 0.22-µm filter (EMD Millipore, Billerica, MA, USA) and stored at 4 °C after confirmation of neutrality in pH.

## Supplementary Information

Supplementary Information 1.

Supplementary Information 2.

Supplementary Information 3.

Supplementary Information 4.

Supplementary Figures.

## Data Availability

The raw CEL files used for the transcriptome analysis in this study were deposited in the NCBI Gene Expression Omnibus database (accession no. GSE149236).
