# Supplementary material for: Inference of transcriptome signatures of Escherichia coli in long-term stationary phase
Source: Sci Rep. 2023 Apr 6;13:5647. doi: 10.1038/s41598-023-32525-4 (PMC10079935; doi:10.1038/s41598-023-32525-4)
Supplement: Supplementary file 5 — Supplementary Figures. [file 41598_2023_32525_MOESM5_ESM.pdf]

Supplementary Figures

Inference of transcriptome signatures of *Escherichia coli* in long-term stationary phase

Sotaro Takano, Hiromi Takahashi, Yoshie Yama, Ryo Miyazaki, Chikara Furusawa and Saburo Tsuru

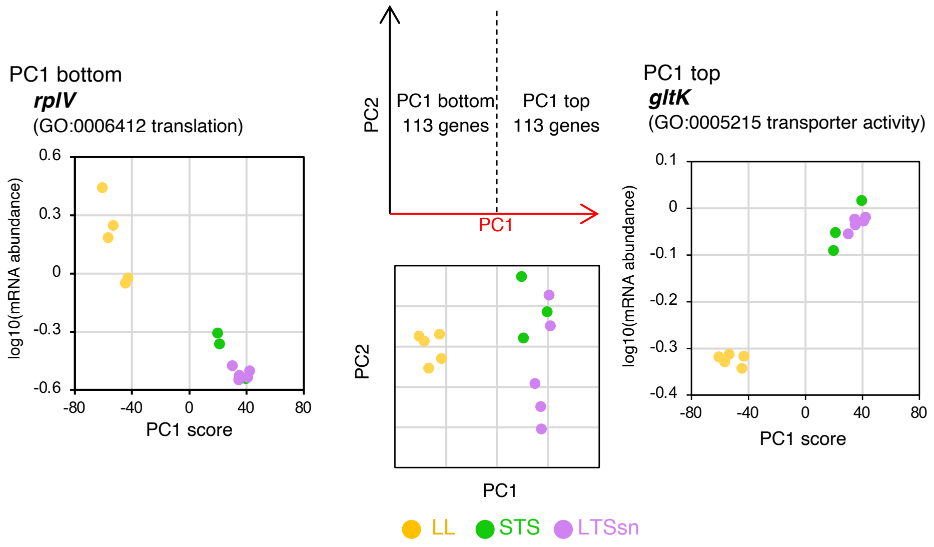

Figure S1. Interrelation of PCA scores and expression levels of extracted genes. The transcription levels of the extracted genes were plotted against the PCA scores. The levels of expression in the members of PC1 top groups show a positive correlation with PC1 scores, whereas the expression levels of the genes in the bottom groups show a negative correlation with PCA scores. Each plot was colored the same as in the case of Fig. 2B.

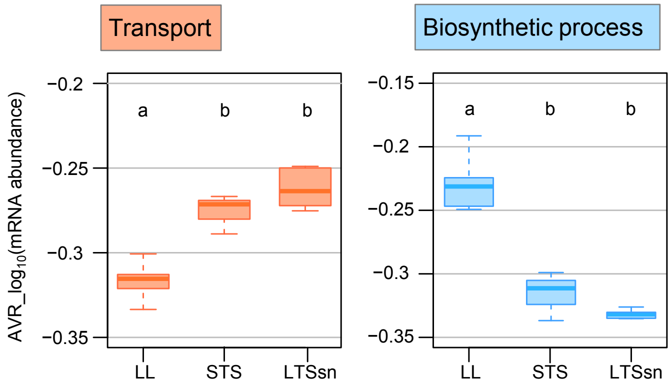

Figure S2. Average mRNA signals in the extracted GO terms. Box plots of average log10 scaled mRNA signals of all members in “transport (GO:0006810),” and “biosynthetic process (GO:0009058),” which largely contribute to PC1 scores in Fig. 2B. Error bars indicate the standard

deviation of biological replicates. Letters above each plot indicate significance groups according to Tukey's HSD test ($p < 0.05$).

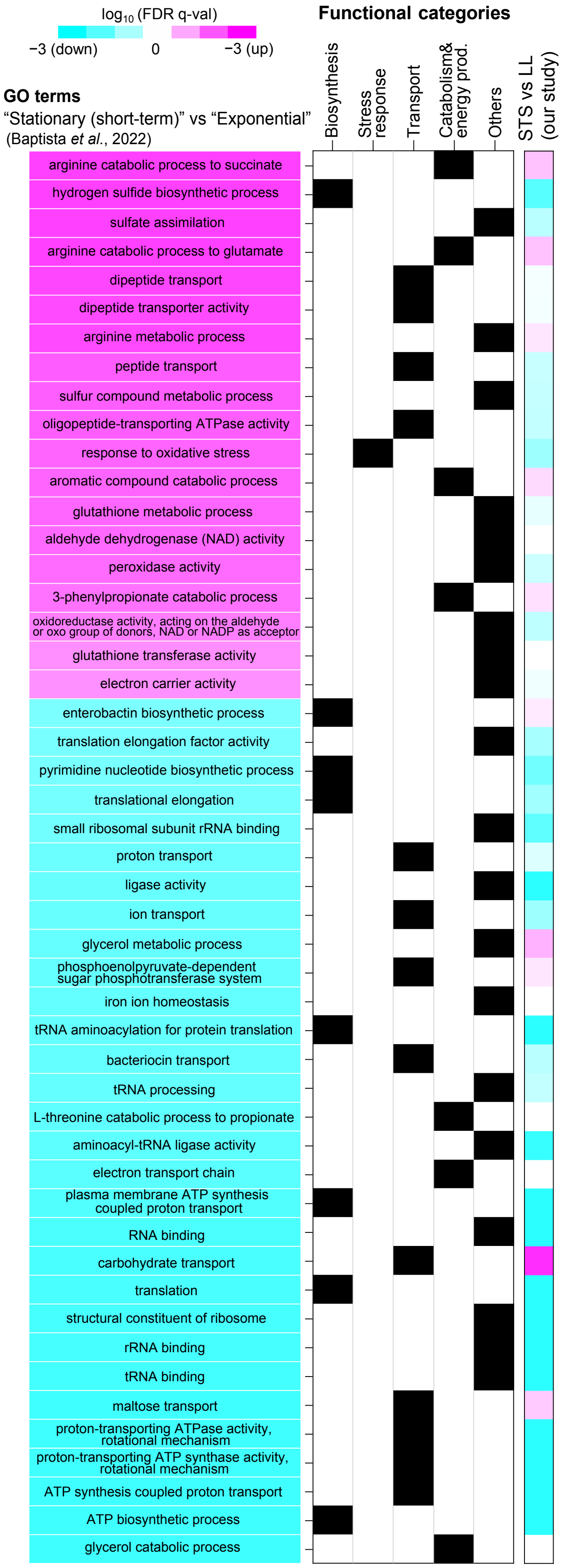

log10 (FDR q-val)
−3 (down)
0
−3 (up)
Functional categories
GO terms
“Stationary (short-term)” vs “Exponential”
(Baptista et al., 2022)
Biosynthesis
Stress response
Transport
Catabolism& energy prod.
Others
STS vs LL (our study)
arginine catabolic process to succinate
hydrogen sulfide biosynthetic process
sulfate assimilation
arginine catabolic process to glutamate
dipeptide transport
dipeptide transporter activity
arginine metabolic process
peptide transport
sulfur compound metabolic process
oligopeptide-transporting ATPase activity
response to oxidative stress
aromatic compound catabolic process
glutathione metabolic process
aldehyde dehydrogenase (NAD) activity
peroxidase activity
3-phenylpropionate catabolic process
oxidoreductase activity, acting on the aldehyde or oxo group of donors, NAD or NADP as acceptor
glutathione transferase activity
electron carrier activity
enterobactin biosynthetic process
translation elongation factor activity
pyrimidine nucleotide biosynthetic process
translational elongation
small ribosomal subunit rRNA binding
proton transport
ligase activity
ion transport
glycerol metabolic process
phosphoenolpyruvate-dependent sugar phosphotransferase system
iron ion homeostasis
tRNA aminoacylation for protein translation
bacteriocin transport
tRNA processing
L-threonine catabolic process to propionate
aminoacyl-tRNA ligase activity
electron transport chain
plasma membrane ATP synthesis coupled proton transport
RNA binding
carbohydrate transport
translation
structural constituent of ribosome
rRNA binding
tRNA binding
maltose transport
proton-transporting ATPase activity, rotational mechanism
proton-transporting ATP synthase activity, rotational mechanism
ATP synthesis coupled proton transport
ATP biosynthetic process
glycerol catabolic process

Figure S3. Comparison with the previous study of gene categories that exhibited significantly different expression levels in short-term stationary (STS) and exponential (LL) phases. We used the expression data from Baptista *et al.*[18], and screened significantly enriched functional categories in higher or lower expressed genes in short-term stationary phase (corresponds to STS in our study) compared to exponential phase (corresponds to LL in our study). We performed GSEA and screened differentially expressed gene categories (False Discovery Rate (FDR), q-value < 0.05). All the screened categories are shown with a heatmap of statistical significance levels (FDR, q-value), where magenta and cyan represent higher and lower expressions in "Stationary (short-term)" than "Exponential" phase respectively. For the comparison to our study, we also performed GSEA for STS and LL in our experiments, and the statistical significance levels in that analysis were shown as a heatmap on the rightmost panel. These screened gene sets were further grouped into five large categories under the same criteria as Fig. 3.

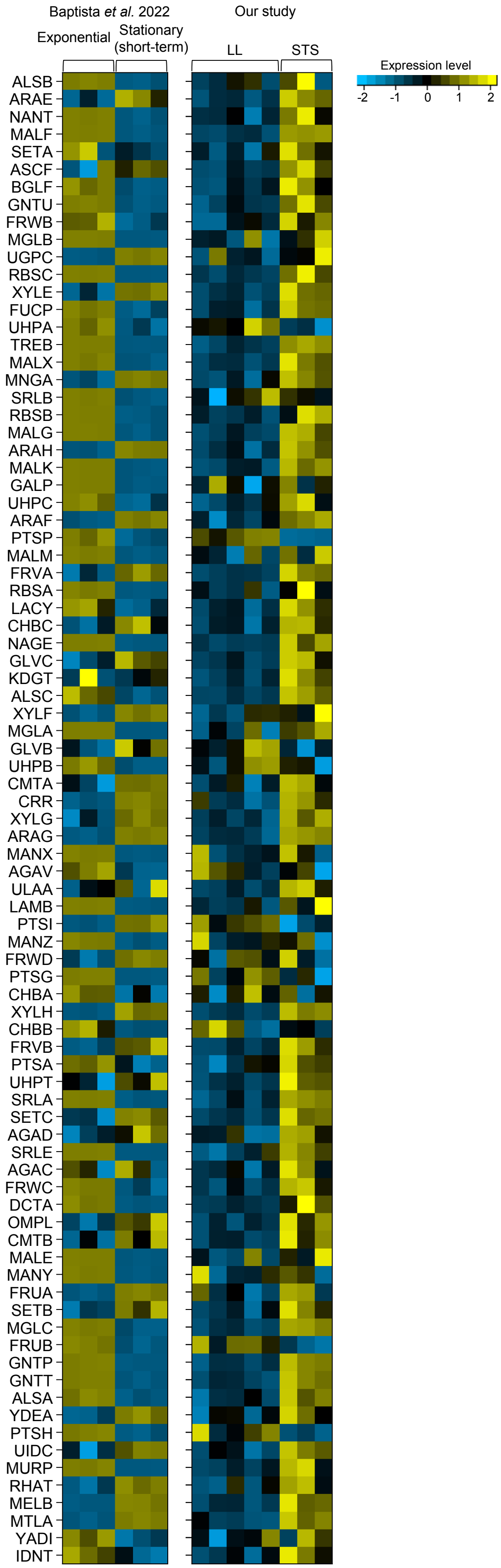
Baptista et al. 2022
Our study
Exponential
Stationary (short-term)
LL
STS
Expression level
-2
-1
0
1
2
ALSB
ARAE
NANT
MALF
SETA
ASCF
BGLF
GNTU
FRWB
MGLB
UGPC
RBSC
XYLE
FUCP
UHPA
TREB
MALX
MNGA
SRLB
RBSB
MALG
ARAH
MALK
GALP
UHPC
ARAF
PTSP
MALM
FRVA
RBSA
LACY
CHBC
NAGE
GLVC
KDGT
ALSC
XYLF
MGLA
GLVB
UHPB
CMTA
CRR
XYLG
ARAG
MANX
AGAV
ULAA
LAMB
PTSI
MANZ
FRWD
PTSG
CHBA
XYLH
CHBB
FRVB
PTSA
UHPT
SRLA
SETC
AGAD
SRLE
AGAC
FRWC
DCTA
OMPL
CMTB
MALE
MANY
FRUA
SETB
MGLC
FRUB
GNTP
GNTT
ALSA
YDEA
PTSH
UIDC
MURP
RHAT
MELB
MTLA
YADI
IDNT

Fig. S4 Expression levels of genes grouped into carbohydrate transport. We showed log10 scaled mRNA levels of genes in “carbohydrate transport (GO:0008643)” in Baptista *et al.*[18] (Exponential: n = 3; Stationary: n =3) and our study (LL: n = 5; STS: n = 3). In individual datasets, the values of mRNA signals in each gene were normalized so that the average and the standard deviation in all replicates are 0 and 1 and shown as a yellow-blue scaled heatmap in biological replicates.

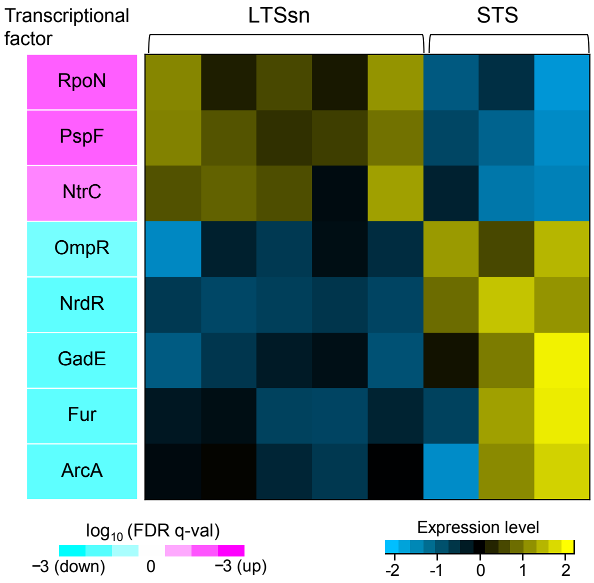

Fig. S5 Differentially expressed gene categories in LTSsn compared to STS in the context of gene regulatory network. Here, we screened transcriptional factors that regulate significantly greater numbers of higher or lower expressed genes in LTSsn compared to STS by GSEA. All screened gene categories by GSEA (False Discovery Rate (FDR), q-value < 0.05) were shown with heatmaps of statistical significance level (FDR, q-value). Magenta and cyan in the heat map mean that the expression level of genes controlled by that transcription factor is higher or lower in LTSsn than in STS, respectively. A yellow-blue scaled heatmap shows the expression levels in each group in biological replicates (LTSsn: n = 5; STS: n = 3) after the normalization in the same manner as Fig.3A.
